# The Happy Life Club™ study protocol: A cluster randomised controlled trial of a type 2 diabetes health coach intervention

**DOI:** 10.1186/1471-2458-11-90

**Published:** 2011-02-09

**Authors:** Colette Browning, Anna Chapman, Sean Cowlishaw, Zhixin Li, Shane A Thomas, Hui Yang, Tuohong Zhang

**Affiliations:** 1 Primary Care Research Unit, School of Primary Health Care, Monash University, Building 1, 270 Ferntree Gully Road, Notting Hill, Victoria, 3168 Australia; 2 Centre of Disease Control and Prevention, Fengtai District Beijing, 3 Xi An Street, Fengtai District, Beijing, 100071 China; 3 Department of Health Policy and Management, School of Public Health, Peking University Health Science Centre, 38 Xueyuanlu Street, Haidian District, Beijing, 100191 China

## Abstract

**Background:**

The Happy Life Club™ is an intervention that utilises health coaches trained in behavioural change and motivational interviewing techniques to assist with the management of type 2 diabetes mellitus (T2DM) in primary care settings in China. Health coaches will support participants to improve modifiable risk factors and adhere to effective self-management treatments associated with T2DM.

**Methods/Design:**

A cluster randomised controlled trial involving 22 Community Health Centres (CHCs) in Fengtai District of Beijing, China. CHCs will be randomised into a control or intervention group, facilitating recruitment of at least 1320 individual participants with T2DM into the study. Participants in the intervention group will receive a combination of both telephone and face-to-face health coaching over 18 months, in addition to usual care received by the control group. Health coaching will be performed by CHC doctors and nurses certified in coach-assisted chronic disease management. Outcomes will be assessed at baseline and again at 6, 12 and 18 months by means of a clinical health check and self-administered questionnaire. The primary outcome measure is HbA1c level. Secondary outcomes include metabolic, physiological and psychological variables.

**Discussion:**

This cluster RCT has been developed to suit the Chinese health care system and will contribute to the evidence base for the management of patients with T2DM. With a strong focus on self-management and health coach support, the study has the potential to be adapted to other chronic diseases, as well as other regions of China.

**Trial Registration:**

Current Controlled Trials ISRCTN01010526

## Background

Over the last decade, chronic illness prevention and management has emerged as a critical issue in the health of populations. The World Health Organisation (WHO) has estimated that cardiovascular diseases (CVD), type 2 diabetes mellitus (T2DM), chronic respiratory diseases and cancers account for 60% of all global deaths [1]. This figure represents 35 million deaths annually and is expected to increase by 17% over the next 10 years [1]. Yet many of these conditions are partly influenced by modifiable risk factors; smoking, sedentary behaviour, low consumption of fruit and vegetables and high alcohol consumption [2]. The WHO have estimated that up to 80% of new cases of T2DM, CVD, and stroke could be prevented by reducing behavioural risk factors and have called for '... interventions to reduce the main shared modifiable risk factors for noncommunicable diseases: tobacco use, unhealthy diets, physical inactivity and harmful use of alcohol.' [1]. The Happy Life Club™, described in this paper, is one such intervention, and utilises health coaches trained in behavioural change principles and motivational interviewing (MI) techniques to address the management of T2DM. Health coaches aim to support participants to improve modifiable risk factors and adhere to effective self-management treatments associated with T2DM. The current paper will present details of the study design and research protocols of the Happy Life Club™ which will be implemented in primary care settings in China.

T2DM is among the most serious health problem affecting the population in China and poses a significant public health issue for the community [3]. As a result of economic development in the past two decades, the Chinese population has experienced marked changes in lifestyle. For example, between 1992 and 2002 the combined prevalence of overweight and obesity increased from 14.6% to 21.8% [4], and physical activity among Chinese adults fell by 32% over the period 1991 to 2006 [5]. These changes have been associated with the increases chronic diseases such as T2DM and CVD [3,4]. The Fourth National Health Service Survey, conducted in 2008 by the China Ministry of Health, revealed that cases of T2DM and hypertension have more than tripled over the previous decade [6]. Recently published data reveal that the age-standardised prevalence of total diabetes (which included both previously diagnosed diabetes and previously undiagnosed diabetes) and pre-diabetes were 9.7% and 15.5% respectively, accounting for 92.4 million adults with diabetes and 148.2 million adults with pre-diabetes [7]. According to the International Diabetes Federation, these latest prevalence figures will mean healthcare expenditure on diabetes in China this year will increase by at least US$1.9 billion, reaching a total of US$6.9 billion [8]. In addition, China has a rapidly ageing population, largely driven by the one child policy, with the associated potential increased burden of chronic diseases [9]. By 2040 around 25% of China's population will be aged 65 years and over [10].

In urban China, the health care system features three levels of health providers: community health, district hospitals, and tertiary teaching hospitals. The majority of outpatient care is provided by Community Health Centres (CHCs) and their affiliated community health stations (61.8%) [11]. Primarily owned and managed by the government, the primary role of these community health services is to provide public health, essential medical treatments, preventive health care, and health education to local communities [12]. On average, Chinese residents access community health services 4.4 times per year, with individuals aged 60+ years accessing community health services 7.3 times per year [13]. Patients with T2DM are predominantly managed by doctors at CHCs and the majority of patients incur out-of-pocket expenses for both medical and pharmaceutical services. However, there is a growing recognition from the Chinese government of the need to adopt best-practice medical management, including the provision of diabetes self-care education and the promotion of healthy lifestyle choices [14].

In most countries, T2DM is managed in primary care settings and clinical practice guidelines recognise the importance of a structured and systematic approach that involves a medical care team, including doctors, nurses and dietitians, as well as patients in the management of the condition [15]. As lifestyle changes are fundamental to the management of T2DM [16], health care teams require skills in both counselling and behaviour change in order to work with the patient to achieve their goals and ensure adherence to recommendations [17]. However skills in these areas are often limited and it has been recognised that training in tailored approaches to counselling, provided by techniques such as MI [18], have the potential to improve lifestyle changes in people with chronic illnesses [19]. MI is a directive, client-centred counselling style for eliciting behaviour change by helping clients to explore and resolve ambivalence [18]. It involves a complex set of skills that grow and develop through disciplined practice with feedback and coaching from a knowledgeable guide [20]. MI has been shown to be effective in improving glycaemic control in adults with T2DM [21]. The Happy Life Club™ uses MI delivered by health coaches to assist patients with diabetes in the management of their condition.

### Development of the Happy Life Club™

The Happy Life Club™ is an adaptation of a diabetes intervention delivered in Australia, the Good Life Club [22]. Patients with T2DM were referred to the Club by their doctor and once enrolled received monthly telephone coaching by a health care practitioner trained in MI and behaviour change techniques. The Happy Life Club™ similarly uses telephone coaching and in addition has face-to-face coaching by nurses and doctors trained in MI and behaviour change techniques. Our evaluation of the Good Life Club revealed that participants preferred to have face-to-face coaching at least in the early phases of the intervention [23].

A pilot study was carried out in five community health stations in the Fangzhuang district of Beijing, China [24]. To assess the feasibility and implementation of the Happy Life Club™ trial design, participants were randomly allocated into either:

1. Intervention group where patients with T2DM received 12 months of health coaching in combination with usual care.

2. Control group where patients with T2DM received usual care only.

In total, one hundred participants (20 per community health station) aged 50 years and over were recruited into the study. Participants in the intervention group received a maximum of 19 telephone coaching sessions and 18 face-to-face coaching sessions over the 12 month period. In the first two months of the intervention, participants received two face-to-face coaching sessions lasting 30 minutes per session and four telephone contacts lasting 20 minutes per session. The frequency of contact decreased over the 12 month period as participants gained skills and confidence in self-management. The pilot study enabled the established trial management procedures, piloted questionnaires, measured compliance and standardised operating procedures. Results from analyses of the pilot study demonstrated that participants in the intervention group had significant improvements in triglyceride levels. Improvements were also made in blood glucose monitoring, adherence to medication, physical activity and psychosocial distress.

### Trial objective

To determine the effectiveness of the Happy Life Club™ intervention in improving the HbA1c level and metabolic, physiological and psychological profiles of participants at 6, 12 and 18 months compared with usual care.

## Methods

### Trial design

The current study is an evaluation of the Happy Life Club™, through an 18-month cluster randomised controlled trial (RCT). Cluster randomised sampling of community health stations was chosen to minimise experimental contamination between the control and intervention participants [25]. The Happy Life Club™ trial is managed jointly by Monash University - Melbourne, Peking University - Beijing and Fengtai Health Bureau - Beijing. The study protocol was approved by Monash University Human Research Ethics Committee.

CHCs will be randomly allocated to one of two groups:

1. Intervention group where patients with T2DM receive 18 months of health coaching in combination with usual care.

2. Control group where patients with T2DM receive 18 months of usual care only.

### Study population & setting

The study will be implemented at CHCs in the Fengtai District of Beijing, China. This district is a large residential community in southwest Beijing with a population of 1.823 million residents and an average life expectancy of 78.7 years [26]. Conveniently located within communities, CHCs deliver primary health care and basic health care to Fengtai residents. The socio-demographic profile of residents in Fengtai varies according to geographic location. West Fengtai is predominantly a regional/rural area; Central Fengtai is a residential suburban area; and East Fengtai is an urban area located near downtown Beijing.

### Recruitment process

**Figure 1:**
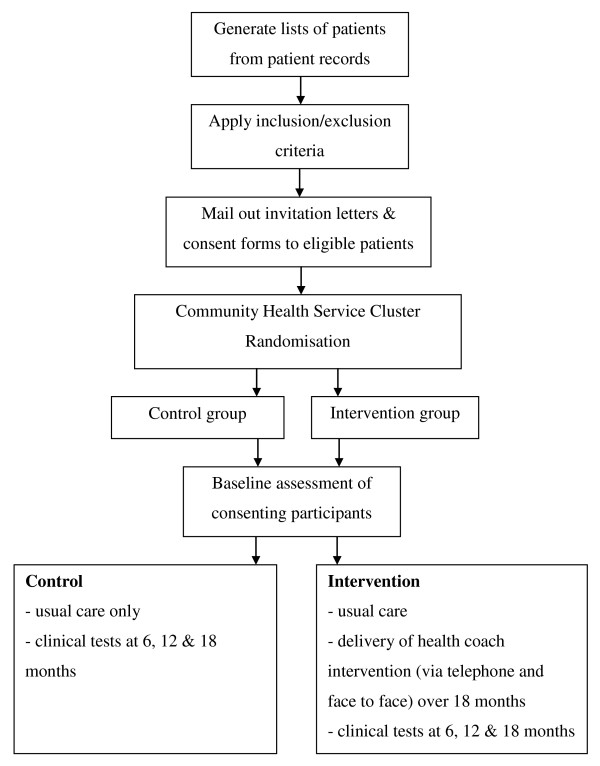
**Flow chart of the recruitment and randomisation of community health centres and participants**.

Figure 1 details the recruitment and randomisation of CHCs and patients into the study. As shown in Figure 1, a list of T2DM patients from each CHC will be generated prior to CHC randomisation, and eligible patients will be contacted by mail.

CHCs will then be randomly assigned to the control (usual care) or intervention group (usual care plus health coaching). Upon recruitment, participants will receive a baseline assessment. All participants will receive clinical assessment at 6, 12 and 18 months.

### Participants

An independent person at each participating CHC will identify eligible participants from the patient health records maintained at each site. Patients with T2DM will be eligible to participate if they are aged 50 years and over, reside in the Fengtai district, have an established health record and are receiving care at one of the participating CHCs. Exclusion criteria include the inability to understand and provide informed consent (e.g., people with cognitive problems), or a medical condition that precludes adherence to recommendations (e.g., end stage cancer, severe mental illness). All eligible participants will be sent an explanatory statement and consent form, either by mail to their home address or in person at their next community health visit. Those wishing to participate in the study will be asked to return the signed consent form to their CHC. All consenting participants will be allocated an ID number will be asked to return to their CHC for the baseline clinical health check. As participation in the trial will require no payment by the participants, we expect that this will provide a high incentive as patients are usually required to pay for CHC visits in China.

### Community Health Centres

As a result of extensive consultation with both the Beijing Health Bureau and Fengtai Health Bureau, it has been agreed that all CHCs located in the Fengtai district will participate in the study, that is, 22 CHCs will be involved in the implementation of the study. An information session will be conducted for all CHC staff, which will thoroughly explain the administrative and implementation aspects of the study. In addition, a member of the research team will visit each CHC to meet with staff and answer specific questions in relation to the study.

### Randomisation

Randomisation will be performed prior to baseline assessment of participants and CHCs will be the unit of randomisation. A member of the research team, blinded to CHC identity, will be responsible for central randomisation. Firstly, CHCs will be stratified by geographical location (east, central, and west) in order to achieve balanced groups according to socioeconomic status. Following stratification, CHCs will be randomly allocated into the control or intervention groups using block randomisation by computerised random allocation software.

### Sample size

Based on conventional methods for estimating a sufficient sample size in a randomised trial with two conditions, and assuming that individuals are randomised to conditions, the number of participants needed is determined mainly by three parameters [27]. These parameters are: (1) the tolerable Type I error rate (assuming the null hypothesis); (2) the desired level of statistical power (assuming the alternative hypothesis); and (3) an anticipated effect size for a relevant test statistic (e.g., standardised mean difference). Consistent with general conventions, [27] the current study specified a 5% Type I error rate as tolerable and required 80% power. The primary outcome measure was glycosylated haemoglobin (HbA1c), measured on a continuous scale, and a recent meta-analysis evaluating psychological interventions to improve glycaemic control in patients with T2DM [21] indicates an expected standardised effect of 0.32 across studies. This was the anticipated effect size estimate specified in the current investigation. Based on these parameters, and using the G*Power 3.1 program [28] to conduct calculations, it was ascertained that a sample size of *n *= 306 (153 per group) would be sufficient to detect an effect size of 0.32 with 80% power, while maintaining 5% Type I error.

As noted, the sample size calculations reported above assume that individuals are randomised to groups. In contrast, cluster randomisation, as used in the current study, is associated with less statistical power; especially when individuals within clusters are similar [29]. This degree of similarity is quantified by the Intra-Class Correlation (ICC) coefficient, which can then be used, along with estimates of the average cluster size (in terms of number of participants per cluster) [30] to calculate an adjusted sample size; inflated to yield the same level of statistical power as if individual randomisation was used [29]. Only a limited number of clusters were available to the current study, and estimates were calculated assuming this fixed number (*k *= 22). As described by Campbell et al. (2000), ICCs for outcome variables in primary care research are generally lower than 0.05, and this value was also specified in the adjusted calculations. A sample size calculator for cluster-randomised trials is available [29] and was used to estimate the required sample size given cluster randomisation. Allowing for a participant attrition rate of 20%, it was estimated a total sample of *n *= 1320 would be required to achieve at least 80% power, while maintaining 5% Type I error. This figure reflects 60 patients per CHC (cluster), and 660 per intervention group.

### Outcome assessment

The trial will run for 18 months and outcome measurements will be assessed at baseline, and again at 6, 12 and 18 months by means of a clinical health check and a self-administered questionnaire. Anthropometric, and blood pressure measurements as well as fasting blood samples, will be taken by independent doctors and nurses from Peking University, who are blinded to group allocation. All participants will be instructed to fast overnight prior to each clinical health check for a minimum of eight hours. Blood samples will be analysed centrally at the CHC Laboratory blinded to group allocation.

#### Physical Examination

1. Anthropometric measurements: Weight will be measured by a beam balance scale and height by a stadiometer attached to a wall. BMI will be calculated as weight (kg) divided by height squared (m^2^) and waist circumference (cm) is measured at the level midway between the lowest rib margin and the iliac crest.

2. Blood pressure: Systolic and diastolic blood pressure will be measured in a sitting position using a mercury sphygmomanometer and measurements will be taken from the right arm. Subjects will be asked to rest for at least 5 minutes before measurements are taken. The first phase of Korotkoff sounds will be recorded as the systolic blood pressure, and the fifth phase as the diastolic blood pressure.

3. Fasting blood samples:

• A Hitachi 7060C Automatic Biochemistry Analysis System will be used to measure fasting plasma glucose, 2 hour postprandial glucose, homocysteine, total cholesterol, triglyceride, and high-density lipoprotein (HDL) cholesterol by standard enzymatic methods. A direct HDL cholesterol method will used.

• Low-density lipoprotein (LDL) will be calculated using the Friedewald formula [31]. LDL calculations will not performed if the triglyceride level is >4.5 mmol/L.

• HbA1c will be measured by a Bayer Healthcare DCA 2000+ analyser.

According to the Chinese Guidelines for Diabetes Prevention and Management [32], the cut-off points of 1.5 mmol/L, 4.5 mmol/L, 2.6 mmol/L, and 1.1 mmol/L will be used in reporting elevated triglycerides, and total, LDL, and HDL cholesterol values. Those having systolic blood pressure (SBP) higher than 130 mmHg and those having diastolic blood pressure (DBP) higher than 80 mmHg will be regarded as having elevated values [33]. Participants with BMI <24 kg/m^2 ^will be regarded as being of normal weight, ≥24 kg/m^2 ^but <28 kg/m^2 ^as overweight, and ≥28 kg/m^2 ^as obese [34,35]. Waist circumference beyond 85cm for men and beyond 80cm for women will be used as the cut-off points for central obesity [34,35].

#### Self Administered Questionnaire

The self administered questionnaire will be completed by participants at each clinical health check. The questionnaire covers the following areas: the participant's background information and socio-economic status, use of health services and health status [36], smoking status [37], alcohol intake [38], psychosocial distress [39,40], quality of life [41,42], diabetes self-care activities [43,44], diabetes management self efficacy [45,46], as well as self rated health which will be measured by SF-1, the first question of the SF-36 [47,48]. All measures used in the questionnaire have previously adapted to suit the Chinese context and have been validated in Chinese populations.

### Intervention

#### Control group

Participants in the control group will receive usual care from their CHC. This may include referral to diabetes specialists, traditional Chinese medicine physicians, and physiotherapists which form part of standard care as outlined in the Chinese Guideline for Diabetes Prevention and Management [32]. Participants in the control group will undergo assessment at baseline, and again at 6, 12 and 18 months. All control group participants will be informed of their results.

#### Intervention group

Participants who are assigned to the intervention group will receive a combination of telephone and face-to-face health coaching in addition to usual care from their CHC. The health coaching will be performed by experienced clinicians (community doctors and nurses). Prior to commencing work on the intervention phase, all health coaches will be required to complete a certified training program in coach assisted chronic disease management. The training program includes the study of key concepts in patient-centred communications, health psychology, epidemiology of key targeted illnesses and conditions, skills training in MI and behaviour change, program evaluation, clinical outcome measurement and the Happy Life Club™ intervention protocol.

The health coaches will aim to assist participants in achieving the treatment targets as outlined in the Chinese Guideline for Diabetes Prevention and Management [32], with the primary goal of treatment of HbA1c of less than 7%. An intervention manual will be used in the study to guide health coaches that utilises existing local guidelines and recommendations (e.g. Dietary Guidelines for Chinese Residents [49]). The initial step in each health coaching session will be to set the agenda for the session with the participant. This will be achieved by simply asking the participant "What would be helpful to talk about today?" Once the participant identifies a key issue for discussion, health coaches will utilise their skills in MI by assessing current behaviour in relation to the issue and exploring the motivation and commitment for change. If a participant displays a need and adequate commitment to change, the health coaches will work with the participant to establish a goal for behaviour change that outlines where, when and how the behaviour change will be performed.

**Figure 2:**
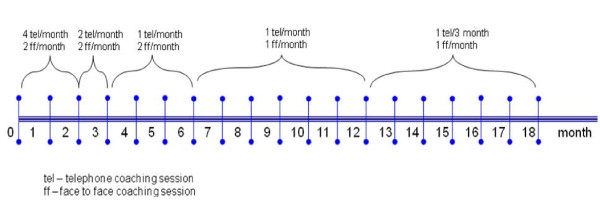
**Frequency of health coaching**.

In the first two months participants will receive two face-to-face and four telephone coaching sessions per month. The frequency of the sessions will decrease over the 18 month intervention period as shown in Figure 2 such that in the last six months of the intervention, participants will receive one face-to-face session per month and one telephone session per three months. This reflects the philosophy of the MI approach whereby over time coach input diminished as the participants gain confidence in self-management. While there is no preset time frame for each health coaching session, it is anticipated from the pilot study that the duration of each telephone session will be approximately 20 minutes, and the face-to-face session approximately 30 minutes. Coaches will record the length of each session.

In addition to health coaching, participants in the intervention group will undergo assessment at baseline, and again at 6, 12 and 18 months. All intervention participants will be informed of their results.

### Statistical analysis

Descriptive statistics will be used to summarise characteristics of both CHCs and participants, with regards to baseline characteristics and patterns of mean change over time. Differences between the intervention and control conditions, on both primary and secondary outcomes, will then be evaluated using multilevel modelling analysis [50]. Multilevel modelling refers to a family of analytic techniques that are appropriate when data has a 'nested' structure, such as when individuals are nested within groups (e.g., patients in hospitals). Repeated measurements represent another type of hierarchical structure, whereby repeated measurements can be viewed as nested within participants. Multilevel modelling techniques, such as multilevel regression, have advantages over alternative methods traditionally used to analyse such repeated measures data (e.g., ANCOVA [51]). For example, multilevel analysis uses data from all available measurements to examine trajectories of change, rather than focussing on a single wave of data such as the final end-point measurement. Furthermore, multilevel analysis can tolerate an 'unbalanced' data-set, whereby participants can have varying numbers of measurements which are irregularly spaced [52]. As such, multilevel analysis does not exclude participants because of missing data or attrition from the study, and is thus consistent with 'intention to treat' principles. Finally, multilevel analysis can be adapted to incorporate more than one type of nesting, as in cluster-randomised trials, where: (1) repeated measurements are nested within participants; and (2) participants are nested within clusters (i.e., CHCs). Multilevel analysis explicitly models, and thus controls for variance at different levels of the hierarchical structure, and has thus been recommended for use with cluster-randomised trials [29].

Analyses will consider participant trajectories over time, and evaluate whether these differ according to levels of exposure to the intervention. Through multilevel analysis, variation in outcome scores will be partitioned across three levels, including: (1) variation in repeated measures within participants, modelled as a function of the initial level (intercept), linear trajectory, and Level-1 residual that may be autocorrelated and heteroscedastic (although different models of the Level-1 error covariance structure can be evaluated [53]); (2) inter-individual variation in the intercept and trajectory, specified as a function of the cluster mean and Level-2 residual assumed to be independently and normally distributed; and (3) variation in cluster means for both intercept and trajectory. The between-cluster variation in trajectory, specifically, is then modelled as a function of an intercept, Level-3 covariate (reflecting exposure to the intervention), and residual. The test statistic for the parameter reflecting experimental condition is used to test the null hypothesis of no treatment effect [54]. One-sided tests will be used in all instances, and a level of p < 0.05 will be used to evaluate statistical significance of the primary outcome. Secondary analysis will use the critical level of p < 0.001 to control Type I error probabilities due to testing across multiple outcomes.

### Qualitative study

Two qualitative studies will be conducted in parallel with the cluster RCT:

1. Participant experience: Focus groups will be undertaken with participants in each arm of the RCT. Purposive sampling techniques will be utilised to ensure a range of selected participants are recruited into the study. We will ensure that the sample includes men and women, age representation, and participants who represent a range of self-management behaviours and self-management skills and confidence levels. The focus group schedule will explore participants' attitudes and experiences of the health coach intervention.

2. Health coach experience: Focus groups will be undertaken with health coaches from each CHC. The focus group schedule will explore the barriers and enablers to the delivery of the intervention and the impact the intervention has had on their professional role. In addition, all health coaches will be completing diaries throughout the intervention. After each coaching session they will record their observation about the session including goals set by the patient and their own reflections on the coaching process. These diaries will be included in the analysis of the qualitative study to evaluate the process of the implementation of the intervention.

Data from the focus groups will be audio-taped, transcribed verbatim, translated into English and entered into NVIVO software [55] to organise the data. To increase rigour, each transcript will be independently coded line by line by two researchers. An inductive process of thematic analysis, as described by Braun and Clarke [56], will be employed to identify key issues and themes within the data.

## Discussion

This cluster RCT has been specifically tailored to the Chinese health care system and has the ability to make a large contribution to the evidence base for the management of patients with T2DM. It is hypothesised that the health coaching intervention delivered in this study will enhance the ability of patients to self-manage T2DM, leading to improved clinical and psychosocial outcomes, thus delaying diabetic complications. In addition, the components of metabolic syndrome and the risk factors for cardiovascular disease will be assessed.

To our knowledge, this is the first trial that combines both telephone and face-to-face health coaching in relation to T2DM management in China. Similar studies have been conducted in other countries, however these have primarily been telephone [57,58] or group based interventions [59,60]. Furthermore, this study will uniquely assess the benefits of intervention delivery for three time periods (6, 12 and 18 months), providing evidence for which is the optimal period of intervention.

This trial is being conducted in a real-world community health setting in China. By implementing the study in community health settings, this study empowers and builds capacity within the existing workforce. This approach embeds the study within community health settings, thereby increasing the generalisability of results [61].

The success of this study is reliant not only on the capacity of the participants to engage in behaviour change but also on the performance of individual health coaches. According to the spirit of MI, the therapeutic relationship is more like a partnership or companionship than expert/recipient roles [18]. It is therefore essential that health coaches are supported in their role. Several approaches have been incorporated into the intervention strategy to ensure coaches receive adequate support: following initial MI training, regular meetings will be scheduled with health coach coordinators; diaries will be kept by each health coach; and a peer support system will be implemented.

An additional challenge for this study will be to ensure that the back-translation of data collected remains an accurate representation of the original version. The use of two, independent, bilingual translators will increase the chances that the original meaning has been retained, will ensure literal accuracy and will help to detect mistakes [62].

This trial, with a strong focus on self-management and health coach support has the potential to be adapted to other chronic diseases, as well as other regions of China. The combination of both quantitative and qualitative methods utilised in this study will allow for a more comprehensive understanding of the implementation process [63], and will provide additional information to guide further research.

## Competing interests

The authors declare that they have no competing interests.

## Authors' contributions

CB & ST led the conception and design of the study and ZL & HY participated. CB & ST obtained research funding for the project. CB & AC led the drafting of the article and all authors contributed to the critical revision. AC & HY will coordinate the data collection. SC will lead the statistical analysis of the data. ZL will coordinate clinician involvement and CB, ZL & ST obtained operational funding for the study. TZ designed the qualitative data collection. All authors will participate in the analysis and interpretation of the trial data. All authors have approved the final version of the manuscript.

## Pre-publication history

The pre-publication history for this paper can be accessed here:

http://www.biomedcentral.com/1471-2458/11/90/prepub
